# Exon sequence requirements for excision *in vivo *of the bacterial group II intron RmInt1

**DOI:** 10.1186/1471-2199-12-24

**Published:** 2011-05-23

**Authors:** Antonio Barrientos-Durán, Isabel Chillón, Francisco Martínez-Abarca, Nicolás Toro

**Affiliations:** 1 Grupo de Ecología Genética, Estación Experimental del Zaidín, Consejo Superior de Investigaciones Científicas (CSIC). C/Profesor Albareda 1, 18008 Granada, Spain; 2 Department of Biochemistry, University of California, 900 University Avenue 92521, Riverside (CA) USA

## Abstract

**Background:**

Group II intron splicing proceeds through two sequential transesterification reactions in which the 5' and 3'-exons are joined together and the lariat intron is released. The intron-encoded protein (IEP) assists the splicing of the intron *in vivo *and remains bound to the excised intron lariat RNA in a ribonucleoprotein particle (RNP) that promotes intron mobility. Exon recognition occurs through base-pairing interactions between two guide sequences on the ribozyme domain dI known as EBS1 and EBS2 and two stretches of sequence known as IBS1 and IBS2 on the 5' exon, whereas the 3' exon is recognized through interaction with the sequence immediately upstream from EBS1 [(δ-δ' interaction (subgroup IIA)] or with a nucleotide [(EBS3-IBS3 interaction (subgroup IIB and IIC))] located in the coordination-loop of dI. The δ nucleotide is involved in base pairing with another intron residue (δ') in subgroup IIB introns and this interaction facilitates base pairing between the 5' exon and the intron.

**Results:**

In this study, we investigated nucleotide requirements in the distal 5'- and 3' exon regions, EBS-IBS interactions and δ-δ' pairing for excision of the group IIB intron RmInt1 *in vivo*. We found that the EBS1-IBS1 interaction was required and sufficient for RmInt1 excision. In addition, we provide evidence for the occurrence of canonical δ-δ' pairing and its importance for the intron excision *in vivo.*

**Conclusions:**

The excision *in vivo *of the RmInt1 intron is a favored process, with very few constraints for sequence recognition in both the 5' and 3'-exons. Our results contribute to understand how group II introns spread in nature, and might facilitate the use of RmInt1 in gene targeting.

## Background

Group II introns are ribozymes and mobile retroelements consisting of a highly structured RNA organized into six distinct domains (dI to dVI). They have a multifunctional intron-encoded protein (IEP) ORF in dIV, encoding a reverse transcriptase-maturase that is required *in vivo *for the folding of the intron RNA into a catalytically active structure [1]. Group II introns splice via a lariat intermediate, in a mechanism similar to that of spliceosomal introns, via two sequential transesterification reactions [1]. In the first step, the 2'-OH group of a branch-point nucleotide residue, usually a bulged adenosine in dVI, attacks the 5' splice junction, resulting in cleavage of the 5' exon and the formation of an intron-3' exon branched lariat intermediate. The released 5' exon remains associated with the intron via base pairing of the intron binding sites (IBS1 and IBS2) to the exon binding sites (EBS1 and EBS2) located in ribozyme domain dI [2]. In the second step, the free 3'-OH of the 5' exon attacks the 3' splice junction, leading to the release of the intron lariat and the ligation of the 5' and 3' exons. Recognition of the 3'exon involves two additional base-pair interactions: the first involves the formation of a single tertiary base-pair between the last position of the intron (γ') and an intron nucleotide (γ) between dI and dII, whereas the second involves the first positions of the exon and specific intron nucleotides. The identity of the intron-binding site in the 3'exon differs between the major classes of group II introns. For IIA introns, the sequence immediately upstream from EBS1 (δ sequence) base pairs with one to three nucleotide residues (δ' position) of the 3' exon for intron splicing (i.e., 3 nt in the *Lactococcus lactis *Ll.LtrB intron). By contrast, most IIB and IIC introns display no statistical evidence of pairing of this δ position with the 3' exon. These group II ribozymes recognize the first nucleotide in the 3' exon, referred to as IBS3, rather than δ', by canonical base-pairing with an intron nucleotide, denoted EBS3 rather than δ, located in the "coordination loop" of dI [3,4]. The δ position is involved in a new tertiary interaction with another residue (denoted δ') also located in the coordination loop [3]. These loop nucleotides are involved in aligning the two exons for the second step of splicing (δ-δ' and IBS3-EBS3 interactions).

The RmInt1 intron of *Sinorhizobium meliloti*, a group IIB intron, is a mobile intron that self-splices *in vitro *in the absence of the IEP [5,6]. Recently, we investigated RmInt1 splicing activity *in vivo *and observed a low efficiency; only 0.07% of exons were joined [7]. This result suggests that bacterial group II introns function more like retroelements than spliceosomal introns. Like other mobile group II introns, RmInt1 recognizes DNA target sequences through both the IEP and base pairing of the intron RNA. Group II IEPs appear to recognize sequences in both the distal 5' and 3' exons, but appear to have no critical nucleotide residues in common. RmInt1 has less stringent requirements for recognition of the distal 5' and 3' exon regions, with T-15 and G+4 the only critical nucleotide residues [8], but is sufficiently long to confer high specificity. The RmInt1 IEP may also interact with nucleotide residues -20 to -16 in the distal 5'-exon, because the elimination of these residues decreases retrohoming efficiency. Despite our knowledge of RmInt1 DNA target-site recognition requirements for reverse splicing and intron mobility, little is known about the exon sequences required for intron excision due to forward splicing.

In this study, we analyzed the nucleotide requirements in the distal 5' and 3' exon regions, EBS-IBS interactions and δ-δ' pairing for excision of the IIB intron RmInt1 *in vivo*. We found that exon recognition by the intron is subject to few constraints, the most critical of which is the EBS1-IBS1 interaction and the δ-δ' pairing. Our findings might have practical implications for the development of RmInt1 as a highly efficient gene targeting vector and support the extraordinary ability of RmInt1 for its dispersal in nature.

## Results and Discussion

### 5' exon nucleotide sequence requirements for RmInt1 excision *in vivo*

**Figure 1:**
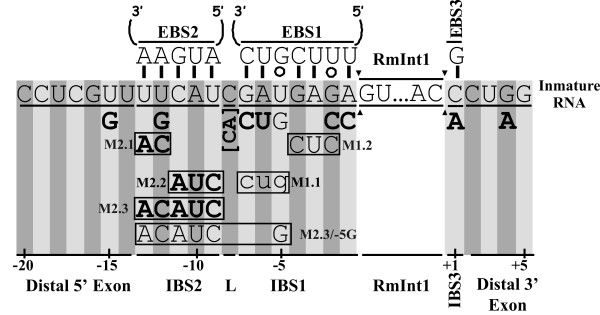
**Nucleotide substitutions in RmInt1 flanking exon sequences**. The schematic diagram shows a wild-type precursor RNA containing the RmInt1 intron and its flanking exons with the predicted EBS/IBS interactions. Arrowheads delimit the RmInt1 intron sequence. Below the flanking exons are indicated all nucleotide substitutions tested and their assignment to three main categories according to the following code: bold upper-case letters, nucleotides and sequences that did not alter the wild-type excision efficiency significantly (86-66% wild-type values); upper-case normal letters, nucleotides and sequences that strongly reduced excision efficiency (to 15-10%); lower-case letters, substitutions that blocked excision (excision products undetectable). Boxed nucleotides correspond to simultaneous substitutions.

Initial studies with the yeast aI5γ group II intron showed that *in vitro trans*-splicing depends on exon-intron binding sequences, the EBS1-IBS1 and EBS2-IBS2 pairings, whereas normal *cis*-splicing requires only the EBS1-IBS1 pairings [9-11]. On the basis of these results, we analyzed the 5' exon nucleotide sequence requirements for RmInt1 excision *in vivo*, by introducing single and multiple nucleotide substitutions into the IBS1 and IBS2 elements (Figure 1) that in the DNA target site blocks intron homing [8]. The linker nucleotide separating the IBS2 and IBS1 elements and the critical position -15 in the distal 5' exon for homing, [8], which has previously been suggested to be involved in a secondary IBS2* element [5], were also analyzed.

**Figure 2:**
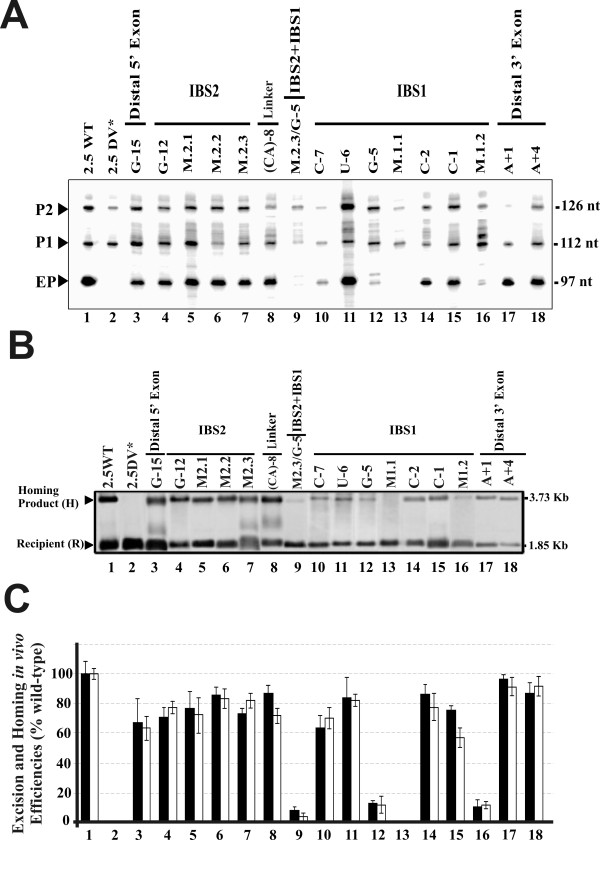
**Effect of exon sequence mutations in intron excision and homing efficiencies**. (**A**) Autoradiograph of the products obtained in a primer extension assay on RNPs isolated from the *S. meliloti *RMO17 strain harboring the wild-type intron pKG2.5 (2.5WT), the excision-defective dV intron mutant pKG2.5D5-CGA (2.5DV*) or the exon mutations generated for this study. The RNP preparations were reverse transcribed with the intron-specific primer, *P*. Three main products were obtained: (EP) a 97 nt cDNA product corresponding to the excised intron RNA; and the (P1) and (P2) cDNA products (112 and 126 nt in size, respectively) that are derived from unspliced precursor transcripts, which appear to be processed at specific 5'U residues as indicated by RACE mapping in the wild-type donor construct. (B) Representative hybridization blot of homing assays. Plasmid pools from *S. meliloti *RMO17 harboring the wild-type pKG2.5 (2.5WT), the excision-defective dV intron mutant pKG2.5D5-CGA (2.5DV*) or the exon mutated intron donors with the pJB0.6LAG target-recipient plasmid were analyzed by *Sal*I digestion and Southern hybridization with an exon-specific probe. The hybridization signals and the size corresponding to the target recipient plasmid (R) and the homing product (H) are indicated. Donor plasmid hybridization signal were removed. (**C) **Bar graph showing the excision (black bars) or homing (white bars) efficiencies, relative to the wild-type, into each of the intron constructs generated, calculated as indicated in Methods. Data are the means of determinations in at least four independent assays with the SD indicated by thin lines.

The excision reaction was characterized by primer extension; with a primer P complementary to a sequence located 80-97 nt from the 5' end of the intron. In these assays, the excised intron RNA was detected as a 97 nt extension product (Figure 2A). In addition to the excised intron lariat, the former product could potentially be generated from either linear intron or lariat-3' exon intermediates. However, we have no evidence that RmInt1 generates excised linear intron molecules *in vivo*: no linear intron RNA was detected by 5'RLM-RACE (RNA ligase-mediated rapid amplification of cDNA 5'ends), primer extension or cRT-PCR (data not shown). Moreover, we showed previously that the lariat form accumulates in the splicing process and that this product is free of lariat-3' exon reaction intermediates Likewise, the RmInt1 splicing process was inefficient, shown by a low detection of ligated exons, more likely to be susceptible to cell degradation [7].

The IBS2 stretch encompasses 5 nt (positions -13 to -9) in the 5' exon of RmInt1 that form Watson-Crick pairs with the EBS2 intron RNA sequence (Figure 1). As shown in Figure 2, the individual replacement, within IBS2, of the U-12 nucleotide by a G residue (lane 4), the combined replacements of the U-13 and U-12 residues with AC (mutant M2.1, lane 5), the replacement of the C-11, A-10 and U-9 nucleotides with AUC (mutant M2.2, lane 6) and complete replacement of the IBS2 sequence UUCAU with ACAUC (mutant M2.3, lane 7) in a construct (pKG2.5) in which the intron is flanked by long stretches of exon sequences (-176/+466), moderately decreased excised intron levels (~71 to 85% of wild-type). A similar result was obtained when the IBS2 sequence was replaced in a construct in which RmInt1 was flanked by short stretches of its natural 5' (-20 nt) and 3' exon (+5 nt) sequences (not shown).

Over the distal 5' region, the replacement of U-15 with a G residue resulted in the retention of ~67% of wild-type intron RNA excision activity (lane 3). The extension of the linker nucleotide between IBS2 and IBS1 by the addition of one nucleotide (C-8 to CA, lane 8) led to a moderate decrease in excision efficiency (~87% of wild-type levels). Thus, in contrast to the critical positions of the EBS-IBS interactions in the DNA Target [8], neither the distal 5'exon region, IBS2-EBS2 nor putative IBS2*-EBS2 [5] pairings are required for intron excision.

By contrast, more heterogeneous results were obtained for nucleotide substitutions within the IBS1 element. The IBS1 stretch encompasses 7 nt, positions -7 to -1 with respect for the intron insertion site, that base-pair with the EBS1 sequence in the intron RNA (Figure 1). Single substitutions at the 5' end, such as the replacement of G-7 with a C residue and of A-6 with a U residue, resulted in excision efficiencies of 63 and 86% those of the wild-type, respectively (Figure 2A and 2B, lanes 10 and 11). Similar results were obtained for individual nucleotide substitutions at the 3' end of the IBS1 sequence: G-2 to C (86.5%, lane 14) and A-1 to C (75.2%, lane 15). By contrast, the replacement of the U-5 nucleotide with a G residue strongly reduced excision to 12% wild-type levels (lane 12), suggesting that this is a critical nucleotide position for cleavage, presumably at the 5' splice site.

Simultaneous changes were also made within the IBS1 element. We focused on simultaneous substitutions in positions -7, -6 and -5 (mutant M1.1, GAU to CUG) that rendered excision undetectable, and simultaneous changes of the -4, -3 and -2 nucleotides (GAG to CUC, mutant M1.2), which strongly reduced intron excision (~13% of wild-type levels). These results identified the IBS1 element in the 5' exon as the most critical for the intron excision reaction. A combined mutant (M2.3/-5G) lacking the canonical IBS2 sequence plus the substitution of the U-5 nucleotides by a G residue in IBS1 rendered intron excision barely detectable (lane 9), which reveals the additional contribution of the IBS2-EBS2 pairing to the efficiency of the intron excision reaction.

### 3' exon nucleotide sequence requirements for RmInt1 excision *in vivo*

In class IIB introns, position +1 at the 3' exon, known as IBS3 (C+1 residue in the RmInt1 3' exon), base-pairs with the EBS3 element in the intron RNA (position 329G), which is located in the "coordination loop" at dI and displaced only one nucleotide from the 3' branch of helix ID^(iv)^[3]. The IBS3-EBS3 interaction has been shown to be important for the second step of splicing [3] and for RmInt1 target-site recognition for intron mobility [8]. Interestingly, the RmInt1 donor construct in which IBS3 C+1 was replaced by an A residue displayed no significant impairment of excision activity (~96% of wild-type activity, Figure 2A and 2B, lane 17). In addition, replacement of the G+4 residue in the 3' exon with an A residue, which has a 40% of reduction on homing efficiency when the target is mutated [8], resulted in an excision efficiency 87% that of the wild type (lane 18). One possible interpretation of our data is that the substitution of IBS3 C+1 by an A residue blocks second step of splicing and the 97 nt extension product is generated from lariat-3'exon intermediates. To solve this question, we performed qRT-PCR and measured exon ligation by the A+1 mutant. As shown in Table 1 the splicing activity of the former mutant was equivalent to that by the wild-type intron. We therefore conclude that neither IBS3-EBS3 pairing nor 3' exon sequences are required for RmInt1 excision *in vivo*.

**Table 1:** RmInt1 splicing activity in *S.meliloti *measured by relative quantification of joined exons in qRT-PCR

Construction	**% splicing ± error**^**a**^
WT	100
DV*	0.76 ± 0.01
+1A	98 ± 1

^**a **^RmInt1 splicing is calculated **by 2**^**-ΔΔ*C***^_**T **_**Method **(21) using qRT-PCR primers and conditions specified in Methods section.

### The excision process is a limiting step for RmInt1 intron mobility

The above results suggest that the RmInt1 excision *in vivo *is a highly favored process, with IBS1-EBS1 pairing the most critical step. As the intron lariat released from the 3'exon remains associated with the IEP, forming a ribonucleoprotein particle complex (RNP) that mediates intron mobility, we investigated whether the observed decrease in excision was correlated with reduced intron mobility. We carried out a homing assay with intron donor and recipient plasmids, in which intron mobility was assessed by DNA hybridization. Once RmInt1 was excised *in vivo *from the exon-mutant donor constructs, the RNPs produced were wild-type. Following introduction of either the wild-type intron (pKG2.5) or mutant constructs into a *S. meliloti *RMO17 strain harboring the target-recipient plasmid pJB0.6as, a broad correspondence was observed between the excision level detected by primer extension and homing efficiency (compare black and white bars in Figure 2C).

### Requirement for δ-δ' interactions in the excision *in vivo *of the RmInt1 intron

**Figure 3:**
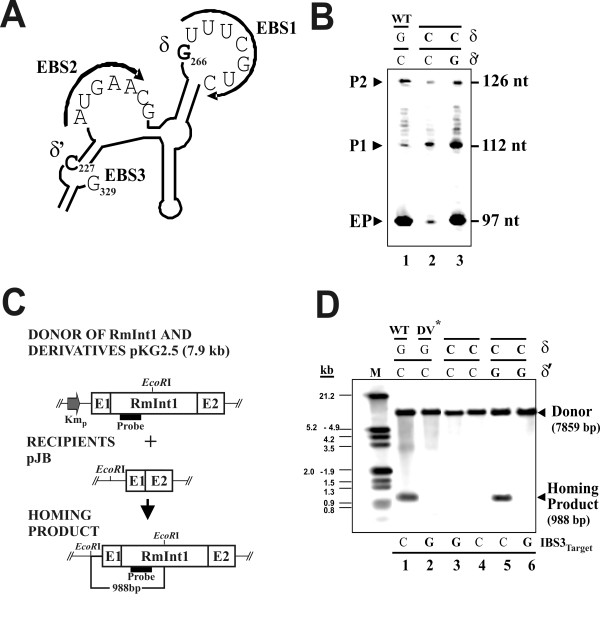
**Study of the role of the δ-δ' interaction in RmInt1 excision *in vivo***. (**A) **Schematic diagram of domain dI focusing on the region containing the EBSs (1, 2 and 3), δ and δ' elements, which are shown in bold typeface. (**B) **Autoradiograph of the products generated in the primer extension assay. Modifications to the δ and δ' wild-type elements in the different mutants are indicated in bold typeface. (C) Diagram of the double plasmid homing assay performed showing Intron donor, recipient plasmids containing RmInt1 target sites (E1+E2), and homing products. Relevant restriction sites and the sizes of DNA fragments used as diagnostic of homing are indicated (**D) **Southern blot obtained in homing assays between derivative donor plasmids (pKG2.5 with modifications of the aforementioned elements) and the wild-type recipient plasmid pJB-20/+5 containing the homing site or a mutant (pJB-20/+5; IBS3 G) shown in bold typeface. An RmInt1 specific probe was used, after the *EcoR*I digestion of plasmid DNA pools for the detection of donor and homing products.

The δ-δ' interaction is thought to be required for the splicing reaction and seems to facilitate base pairing between the 5'exon and the intron; disruption of this interaction interferes with exon binding [3]. In the case of RmInt1, this interaction involves residues 266G (δ) and 227C (δ') (Figure 3A). We found that the disruption of this pairing in the RmInt1 intron (Figure 3B), through the replacement of the δ residue G266 nucleotide with a C residue (δC) strongly decreased excision (to ~8% of wild-type levels), whereas the introduction of a compensatory δ'C to G mutation (δ-δ', C-G instead of the wild-type G-C pairing) restored excision to the level of the wild-type intron (lanes 2 and 3, respectively). Consistent with the lower excision efficiency observed following disruption of the δ-δ' interaction (δC mutant), mutant mobility was undetectable at both the wild-type DNA target site and a mutant target site at which the IBS3 nucleotide +1C was replaced with a +1G nucleotide (Figure 3C, lanes 3 and 4). As expected, the introduction into the δC mutant of the compensatory δ'C to G mutation to restore δ-δ' pairing, (C-G instead of the wild-type G-C pairing) re-established mobility at the wild-type target (lane 5), but not at the mutant target carrying a G nucleotide at position +1 (lane 6). These results provide evidence for the occurrence of intradomain dI canonical δ-δ' pairing *in vivo *and its importance for RmInt1 excision.

## Conclusions

We show here that the intron-exon interaction IBS1-EBS1 and δ-δ' interaction in the domain dI of the rybozime, play an important role in the RmInt1 excision *in vivo*. By contrast, however, IBS2, residue U-15 in the distal 5' exon and IBS3 or G+4 in the 3' exon are not required for the excision reaction, despite the requirement of these elements for the homing process. Nucleotide replacements causing a moderate or severe decrease in the excision efficiency of the intron displayed a similar decrease in homing efficiencies, probably due to a decrease in the production of RNPs. Thus, RmInt1 has different exon nucleotide sequence requirements in either both forward (intron excision) and reverse (intron retrohoming) splicing reactions. Our results contribute to understand how group II introns spread in nature, and might facilitate the use of RmInt1 in gene targeting.

Group II introns can move site-specifically to homologous intronless genes, in a process known as retrohoming or, at much lower frequencies, to novel ectopic sites, in a process known as retrotransposition. It is believed that the latter process has led to the wide dispersal of mobile group II introns among bacterial species and also may have been used to invade eukaryotic genomes, where mobile group II intron are thought to have evolved into spliceosomal introns and non-long-terminal-repeat retrotransposons [12]. Intron insertion-sites with good EBS/IBS pairings suggest insertion via retrohoming, whereas poorly matched IBS sequences suggest insertion via retrotransposition. It is generally accepted that only those introns inserted by retrohoming at sites with complementary IBS sequences are expected to splice efficiently. However, we show here that RmInt1 excision *in vivo *is subject to few constraints, the most critical of which is the EBS1-IBS1 interaction. Thus, the spread of group II introns into bacterial genomes from ectopic sites to homing sites may occur efficiently, increasing their ability to spread in nature.

Gene targeting technology based on group II introns requires the modification of IBS2, IBS1 and IBS3, to render these sites complementary to the retargeted EBS2, EBS1 and EBS3 sequences for efficient RNA splicing [13,14]. We have recently demonstrated that the RmInt1 intron could be used for gene targeting, following the design of introns for insertion into different target sites [15]. As the predicted base-pairing interactions with the RmInt1 intron RNA -- EBS2-IBS2 and EBS3-IBS3 -- have only a small effect on overall intron-splicing efficiency, we hypothesize that the provision of IBS2 and IBS3 nucleotide combinations complementary to modified intron EBS2 and EBS3 sequences in intron plasmid constructs may not be necessary, potentially facilitating the use of RmInt1 in gene targeting.

## Methods

### Bacterial strains, media and growth conditions

Rhizobial strain *S. meliloti *RMO17, lacking the RmInt1 intron, was grown at 28°C on TY or defined minimal medium [16]. *E. coli *DH5α was used for cloning and the maintenance of recombinant plasmids; it was cultured at 37°C in Luria-Bertani medium [17]. Antibiotics were added to the medium when required, at the following concentrations: kanamycin, 200 μg/ml for *S. meliloti *and 50 μg/ml for *E. coli*; ampicillin, 200 μg/ml and tetracycline, 10 μg/ml.

### RmInt1 and its intron derivatives

RmInt1 donor derivatives containing point or accumulative substitutions in the flanking regions of intron insertion sites and ribozyme domain dI, including the δ and δ' sequences, were generated with the *Altered Sites II in vitro Mutagenesis System *(Promega, Madison, WI), using pAL2.5 as a template. This plasmid was generated by inserting a 2.5 kb *Sph*I fragment containing the entire RmInt1 sequence and flanking exons (-174/+466) from pGEmex2.5 [18] into the pALTER-1 vector. Nucleotide substitutions were checked by sequencing and the mutated 2.5 kb DNA fragment was retrieved from pAL2.5 as a *Bam*HI/*Spe*I fragment and inserted into pKG0 (pKG2.5 and mutant derivatives), which constitutively expresses RmInt1 and its derivatives under control of the promoter of the Km resistance gene [18].

### RNP isolation

RNPs were isolated essentially as previously described [19,20]. RNPs were isolated by picking a single colony from a plate of each bacterial strain and using it to inoculate 3 ml of TY medium, which was then incubated at 28°C for two days, allowing the cells to reach stationary phase. We then added 1 ml of the culture to 200 ml of TY medium in a 500 ml flask and incubated the flask at 28°C until the culture reached the exponential growth phase (OD_600 nm _of 0.6 to 0.9).

### Primer extension assays

These experiments were carried out on RNP preparations, since the lariat intron seems to be less susceptible to degradation than ligated exons, due to its association with the IEP forming ribonucleoprotein particles (RNPs). Primer extension reactions were essentially carried out as previously described [20]. Samples were then resolved by electrophoresis in a denaturing 6% polyacrylamide gel. The cDNA bands corresponding to the resolved extension products were quantified with the Quantity One software package (Bio-Rad Laboratories). We assume that the generated mutations in the exons do not affect intron RNA stability, which is mainly affected by its interaction with the IEP. Thus, the excision efficiency was calculated as the ratio of the EP (97 nt) product from each intron construct to wild-type construct, normalized with respect to the sum of P1 and P2 (112 and 126 nt) unspliced precursor products and expressed as a percentage.

### qRT-PCR

For the quantification of ligated RmInt1 flanking exons, a 283 bp fragment was amplified with 3'RTSp (5'-ATTCAACGGGAAACGTCTTG) and +9 (5'-AGCGGCGCCATGTTCGTCTT) primers. To normalize the loading amount of the starting material, a 500 bp fragment of the 16S ribosomal subunit RNA was amplified with *S.meliloti *specific primers Sino 452 (5'-CGTTGTTCGGAATTACTGG) and Sino 952 (5'-TGTCTCCGATCCAGC). Primer specificity was determined by melting-curve analysis and gel electrophoresis (Additional File 1). We conducted the qRT-PCR analysis by using the iCycler System (Bio-Rad, Additional File 2). Each reaction was run in triplicates and contained 5 μl of cDNA template (equivalent to 500 ng total RNA), 0.5 mM dNTP, 5 pmol of each oligonucleotide primer, 7.5 mM MgCl_2_, 2.5 μl of a 1:10,000 dilution of SYBR Green, 2.3 μl of reaction buffer and 0.5 U of Platinum *Taq *DNA Polymerase (Invitrogen), with the final volume made up to 23 μl with DEPC water. The PCR cycling conditions were as follows: hot start, with heating at 94°C for 3 min, followed by 40 cycles of denaturation at 94°C for 30 s, annealing at 55°C for 30 s, and extension at 72°C for 30 s. Controls without a template were also included. Two independent RNA preparations from each strain were tested. Relative quantification was carried out for splicing, based on the 2^-ΔΔCT ^method [21]. Briefly, RmInt1 splicing is defined by the following equation: 2^-ΔΔCT ^where ΔΔCT = ΔCT (DV* or +1A) - ΔCT (WT). ΔCT (DV* or +1A) = CT (DV or +1A mRNA amplified by 5-6 primers) - CT (DV or +1A mRNA amplified by *S. meliloti *specific 16S primers Sino 452 and Sino952). ΔCT (WT) = CT (WT mRNA amplified by 5-6 primers) - CT (WT mRNA amplified by *S. meliloti *specific 16S rRNA primers Sino 452 and Sino952). The result is expressed as a percentage related to WT construction. The error is calculated applying the formula: 2^-ΔΔCT ^· ln2 · ΣSE.

### Homing assays

Double-plasmid homing assays were performed using the *S. meliloti *RmInt1-less strain RMO17. We studied homing between compatible intron donor (pKG2.5 and mutant derivatives) and target-site recipient plasmids (pJB0.6as (-174/+466) or pJB-20/+5) as previously described [22]. Plasmids were mobilized from *E. coli *DH5α to rhizobial strains by tri-parental mating, using pRK2013 as a helper plasmid [23]. Plasmid pools were isolated from exponentially growing cultures in TY of four to ten transconjugants digested with the indicated restriction enzymes and resolved by electrophoresis in 0.8% Tris-acetate agarose gels and vacuum blotted onto nylon filters according to the manufacturer's indications (Roche Diagnostics GmbH, Mannheim, Germany). DNA hybridization was performed under high-stringency conditions, with DIG-labeled probes generated by PCR and specific for the IS*Rm2011-2 *element (spanning positions 562-1025; used in Figure 2) and the RmInt1 group II intron ribozyme (spanning positions 147-594; used in Figure 3). Hybridizing bands corresponding to homing products (H) and recipient plasmids (R) were analyzed with Quantity One software (Bio-Rad Laboratories) and invasion efficiency of each target site calculated as 100[H/(H+R)] [18].

## Authors' contributions

AB-D carried out the molecular genetic studies and drafted the manuscript. IC carried out the qRT-PCR assays. FMA and NT conceived and participated in the design of the study. In addition, NT coordinated the study and supervised the drafting of the manuscript. All authors have read and approved the final manuscript.

## Supplementary Material

Additional file 1 **Specificity of primer pairs used in real-time PCR**. A 40-cycle end-point PCR reaction was carried out with primers 3'RTSp and +9 to amplify ligated RmInt1 flanking exons and with primers Sino 452 and Sino 952 for amplifying the 16S ribosomal subunit as a normalizer of the amount of RNA. Click here for file

Additional file 2 **Amplification plot**. Fluorescence was plotted as a function of the PCR cycle number using iQ5 software package (Bio-Rad). Click here for file
