# Supplementary figures and images for: Exon sequence requirements for excision in vivo of the bacterial group II intron RmInt1
Source: BMC Mol Biol. 2011 May 23;12:24. doi: 10.1186/1471-2199-12-24 (PMC3123198; doi:10.1186/1471-2199-12-24)

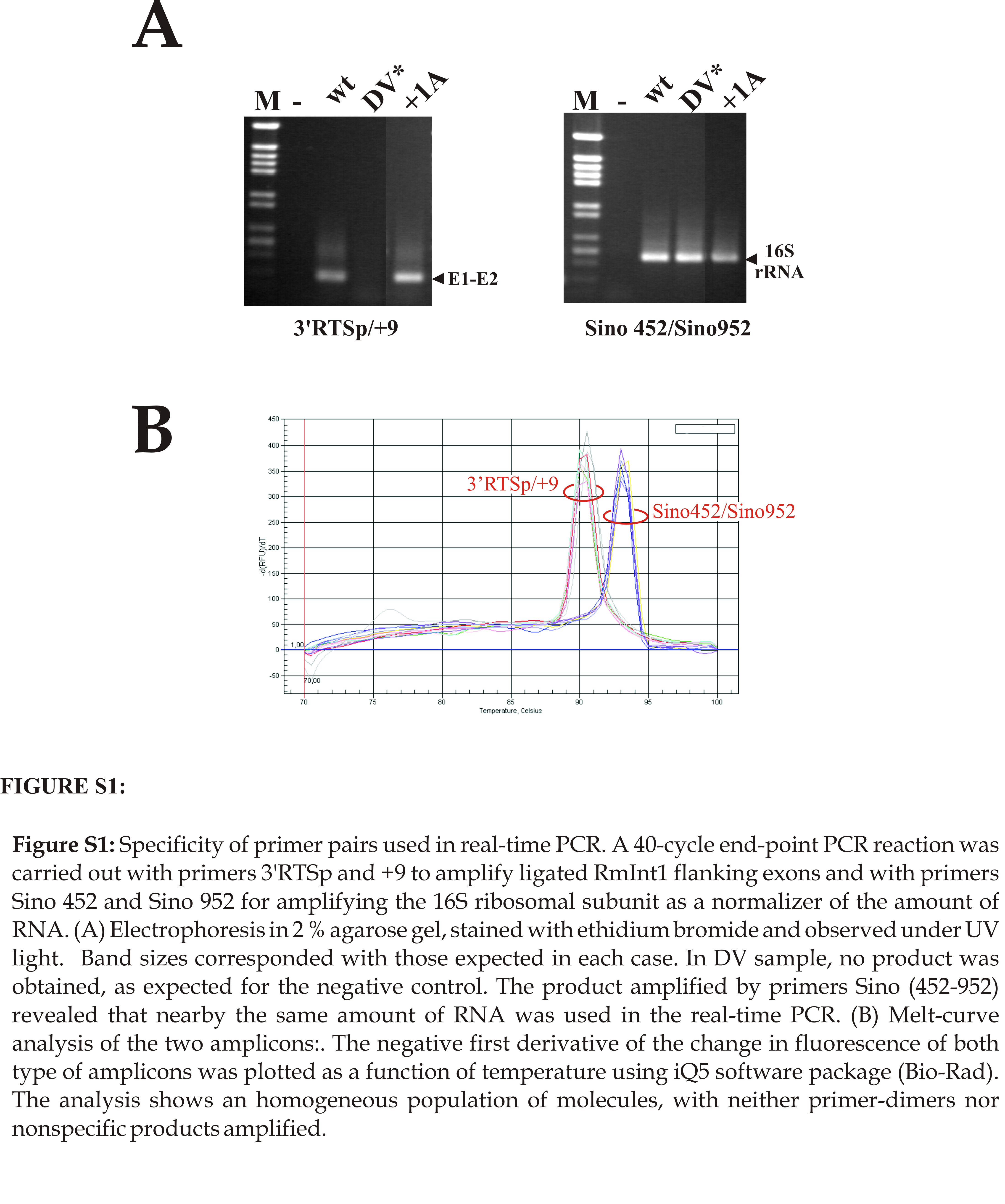

Supplement: Additional file 1 — Specificity of primer pairs used in real-time PCR. A 40-cycle end-point PCR reaction was carried out with primers 3'RTSp and +9 to amplify ligated RmInt1 flanking exons and with primers Sino 452 and Sino 952 for amplifying the 16S ribosomal subunit as a normalizer of the amount of RNA. [file 1471-2199-12-24-S1.TIFF]

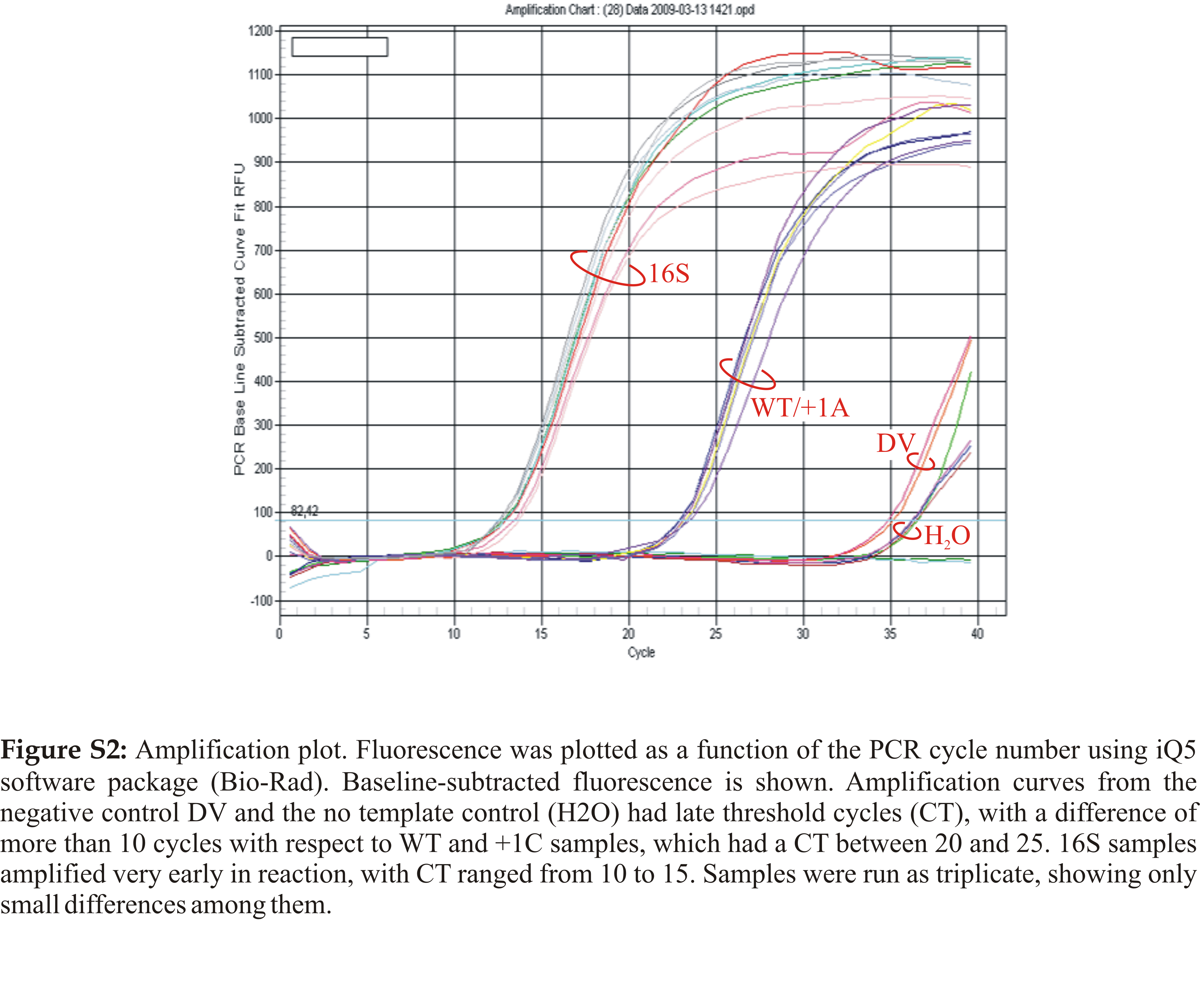

Supplement: Additional file 2 — Amplification plot. Fluorescence was plotted as a function of the PCR cycle number using iQ5 software package (Bio-Rad). [file 1471-2199-12-24-S2.TIFF]
